# Plasma Cell Granuloma Mimicking Plasmacytoma Illustrated by ^18^F-Fluorodeoxyglucose Positron Emission Tomography

**DOI:** 10.3390/hematolrep18020022

**Published:** 2026-03-17

**Authors:** Osamu Imataki, Hiroaki Ide, Akihiro Takeuchi, Makiko Uemura

**Affiliations:** 1 Department of Laboratory Medicine, Kawasaki Medical School, General Medical Center, 2-6-1 Nakasange, Kita-District, Okayama City 700-8505, Okayama, Japan; 2 Division of Hematology, Department of Internal Medicine, Faculty of Medicine, Kagawa University, 1750-1 Ikenobe, Miki-Town 761-0793, Kagawa, Japan; uemura.makiko@kagawa-u.ac.jp; 3 Division of Hematology, Department of Internal Medicine, Takamatsu Municipal Hospital, 847-1 Busshouzan-cho-kou, Takamatsu City 761-8538, Kagawa, Japan; hiroide31@yahoo.co.jp; 4 Division of Clinical Laboratory, Kagawa University Hospital, 1750-1 Ikenobe, Miki-Town 761-0793, Kagawa, Japan; takeuchi.akihiro@kagawa-u.ac.jp

**Keywords:** plasma cell granuloma, plasmacytoma, infectious aortitis, positron emission tomography, ^18^F-fluorodeoxyglucose

## Abstract

**Background:** Plasma cell granuloma is generally considered a pseudotumor formed by reactive, polyclonal plasma cells. Although most cases can show polyclonal gammaglobulin production, quite a minority may exhibit monoclonal gammopathy, which mimics plasma cell neoplasms such as multiple myeloma or plasmacytoma. Because of this overlap, distinguishing reactive monoclonal proliferation from true malignancy is clinically essential. **Case report:** A 79-year-old man was presented with an anterior chest wall mass that had grown during investigation for fever of unknown origin. ^18^F-fluorodeoxyglucose positron emission tomography (FDG-PET) revealed a sternal bone mass (SUVmax 9.04), aortic uptake of bifurcation (SUVmax 7.08), and Th7/8 soft tissue mass (SUVmax 5.32). Results from the FDG-PET revealed infectious reactions. A chest wall biopsy revealed high degree proliferation of plasma cells. Hematologists suspected plasmacytoma. The pathologist did not diagnose plasmacytoma; thus, there remains a possibility of reactive granuloma lesion. Lastly, the patient’s vertebral soft tissue mass culture yielded Staphylococcus aureus. The patient was treated with antimicrobials and responded well. **Discussion:** In the presented case, FDG-PET revealed an aortic mass with an aortic aneurysm, a sternal mass, and a vertebral mass, as multiple lesions. The abscess lesions that initially resembled multiple plasmacytomas were identified as plasma cell granuloma. The final diagnosis required demonstrating biopsy and definitive monoclonality. Light-chain restriction or monoclonal protein should be considered in the clinical context. Ultimately, this case highlights the diagnostic value of FDG-PET and the importance of differentiating reactive plasma cell granuloma from true plasma cell neoplasm to guide appropriate management. **In conclusion**, a reactive plasma cell granuloma associated with infectious aortitis can exhibit monoclonal gammopathy, mimicking plasma cell neoplasm. Careful pathological and clinical evaluation is essential to avoid misdiagnosis and ensure proper treatment.

## 1. Introduction

Plasma cell granuloma is a pseudotumor made up of reactive plasma cells directing polyclonal nature [1]. This granuloma produces polyclonal gammaglobulin. However, some cases show monoclonal gammopathy [1]. Still, monoclonal gammaglobulin does not always equal malignancy. Chronic infections, autoimmune diseases, and inflammatory pseudotumors can occasionally show restricted light-chain expression. This phenomenon is observed as reactive monoclonal gammopathy or monoclonal immunoglobulin production in inflammatory lesions. Clinically, it can arise in virtually any organ, producing symptoms that depend entirely on location rather than a uniform systemic pattern. The organ distribution of plasma cell granuloma is various. Plasma cell granuloma can occur in any organ, though classic sites including lungs as the most common site, brain, kidney, stomach, heart, and oral cavity. The clinical presentation of plasma cell granuloma is location-dependent. Pulmonary lesions are solitary pulmonary nodules, cough, chest pain, or incidental radiographic findings. Lesions may be locally invasive.

Pathologically, plasma cell granuloma is a non-neoplastic inflammatory lesion predominantly composed of polyclonal plasma cells within a background of storiform fibrosis and spindle-cell proliferation. In some cases, it is considered to be part of the IgG4-related disease spectrum. However, it is important to distinguish these cases from plasma cell neoplasms, such as multiple myeloma and plasmacytomas. Diagnostic confusion is not uncommon because plasma cell granuloma can resemble malignancy clinically and radiologically. A diagnostic indicator of plasma cell granuloma is the proliferation of reactive plasma cells in the absence of monoclonal gammaglobulinemia. Conversely, a determinant of a plasma cell neoplasm is a monoclonal plasma cell proliferation manifesting monoclonal gammaglobulinemia. But what if plasma cell granuloma were concomitant with monoclonal gammopathy of undetermined significance? Oligoclonal gammopathy can be observed in reactive inflammatory diseases such as plasma cell granuloma, which are not caused by plasma cell neoplasms. We encountered a case of infectious aortitis with monoclonal gammopathy and plasma cell granuloma.

Since plasma cell granuloma can clinically and radiologically mimic malignant tumors, establishing an accurate preoperative diagnosis remains challenging. ^18^F-fluorodeoxyglucose positron emission tomography (FDG-PET) has been increasingly utilized in the evaluation of inflammatory pseudotumor including plasma cell granuloma, as these lesions often demonstrate variable but sometimes intense FDG uptake, leading to potential diagnostic confusion with malignancy [2,3]. Several case reports have described FDG-avid pseudotumors in diverse anatomical sites, including the colon, spleen, kidney, liver, and head and neck regions [3,4,5,6], highlighting both the utility and limitations of FDG-PET in differentiating benign inflammatory masses from neoplastic processes. However, evidence remains limited, and characteristic metabolic patterns have not been fully established. In this context, the present case contributes additional insight into the FDG-PET features of plasma cell granuloma and underscores the importance of considering inflammatory pseudotumor in the differential diagnosis of FDG-avid lesions.

## 2. Case Report

**Figure 1:**
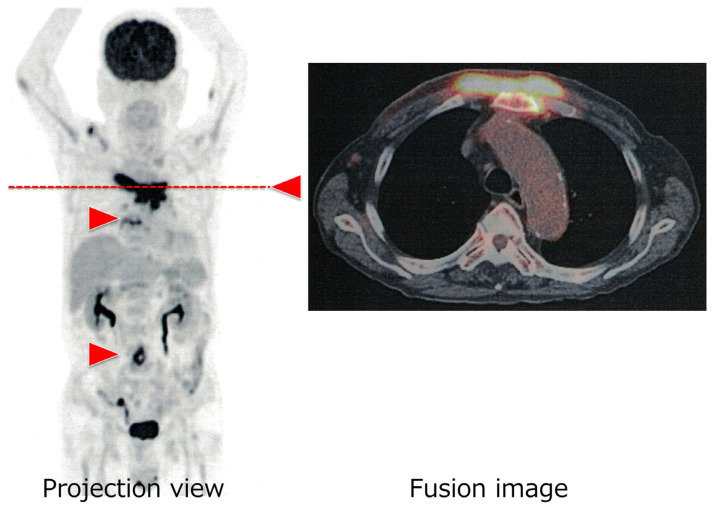
The patient’s ^18^F-fluorodeoxyglucose positron emission tomography. The red arrowheads indicate the focal lesions in the patient: a chest wall mass of sternal bone (SUVmax 9.04), a mass at the aortic bifurcation (SUVmax 7.08), and a soft tissue mass at Th 7/8 (SUVmax 5.32). The right panel shows a transverse image of the fusion CT scan, which shows the accumulation of ^18^F-FDG PET at the level of the red dashed line. A three-dimensional PET scan was performed one hour after an intravenous injection of 4.0 MBq/kg of ^18^F-FDG. Count-equivalent images were obtained for the standard acquisition (two min for bed position).

We present a 79-year-old patient who was referred to our division with a history of weeks of fevers and fatigue, with a recently growing anterior chest wall mass, during evaluation for fever of unknown origin. The patient had a history of diabetes mellitus and hypertension. Both diseases were being managed by his community clinic. The patient was further investigated for the cause of fever by repeated culture studies, infectious biomarkers, and computed tomography (CT). Examination for more than 2 months failed to identify the fever’s focal point. During the research period, chest wall mass increased. Then, the patient was referred to our institute to diagnose the mass. The initial laboratory examination (Table 1) revealed mild inflammatory response with hypergammaglobulinemia. This implied chronic infectious disease or reactive/primary gammopathy. To identify the mass localization, ^18^F-fluorodeoxyglucose positron emission tomography (FDG-PET)/CT was conducted. The FDG-PET/CT revealed a chest wall mass derived from sternal bone (7.0 cm in diameter) with FDG uptake (SUVmax 9.04), uptake of the aortic bifurcation (SUVmax 7.08), and soft tissue mass at Th 7/8 (SUVmax 5.32) (Figure 1). The FDG-PET result suggested infectious reactive lesions.

An immunological study of the patient showed that levels of IgA, IgG, and IgM were intact or increased to 433, 2103, and 330 mg/dL, respectively, without unbalanced paraproteinemia. The patient’s bone marrow examination revealed an increase in plasma cells (6.0%), and abnormal clones were scarcely found among those plasmacytes. The chromosomal analysis revealed no abnormal karyotype. A trace amount of IgG-λ type monoclonal protein was found in the patient’s serum. As a diagnostic study for infectious diseases, two sets of blood culture were all negative. Other microbiological studies including influenza virus antigen, SARS-CoV-2 antigen, urinary pneumococcal antigen, and urinary Legionella antigen were all negative. Clonality was defined phenotypically.

**Figure 2:**
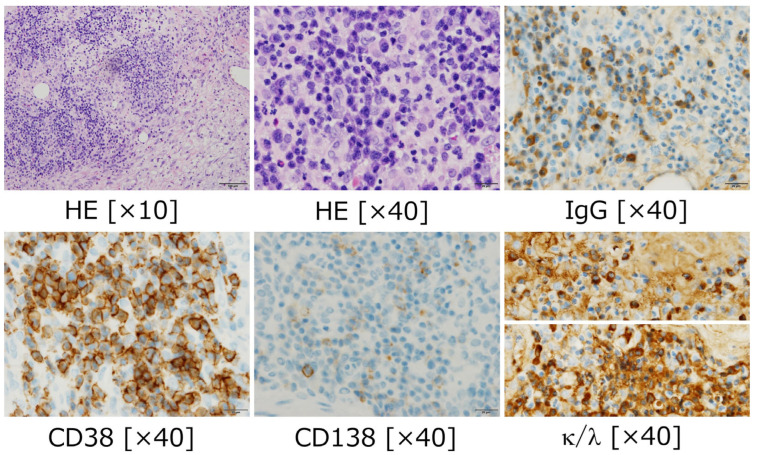
The patient’s pathology of the chest wall. Plasma cells infiltrated and proliferated with various inflammatory cells including eosinophils, neutrophils, and lymphocytes. Plasma cells were positive for IgG and CD38; however, the light-chain staining (κ and λ) were both strongly positive without apparent predominancy. This pathological diagnosis could not conclude a monoclonal proliferation of plasma cells.

A biopsy of the chest wall mass showed a high degree of plasma cell proliferation (Figure 2), prompting the hematologists to suspect plasmacytoma. However, the pathologist did not diagnose plasmacytoma; thus, there remains a possibility of reactive granuloma lesion. Finally, the patient’s vertebral soft tissue mass culture yielded *Staphylococcus aureus*. The patient was treated with antimicrobials and clinical conditions tuned well.

## 3. Discussion

Infectious and non-infectious aortitis may be caused by various distinct diseases. Such a variety of diseases included: systemic infection of tuberculosis, non-tuberculosis mycobacterium, syphilis, auto-immune diseases, Takayasu aortitis, IgG4-related disease, and giant cell aortitis [7]. Infectious aortitis is a rare clinical condition that is most commonly associated with an abdominal aortic aneurysm [8]. FDG-PET is a powerful imaging modality to identify and display vascular lesions including aortitis [9,10]. Radiographical imaging can show an initial screening of the cause of aortitis [11]. The most effective methods for locating and diagnosing aortitis are FDG-PET [12]. Plasma cell granulomas have been observed involving numerous organs or tissues [1]. Radiological imaging techniques can be used to detect inflammatory pseudotumors in different parts of the body [13]. The patient was finally discovered to have a bifurcated aortic aneurysm of infectious aortitis, as well as a sternal growing mass forming plasma cell granuloma and vertebral abscess. Such various tissue of plasma cells infiltration in multiple lesions implied multiple plasmacytoma in clinics. To determine the final confirmation, pathological evidence is essential. We emphasize the diagnostic limitations of plasma cell granuloma from monoclonal gammopathy in inflammatory conditions.

Plasma cell granuloma and plasma cell neoplasm can look deceptively similar clinically and microscopically but are fundamentally different in biology, behavior, and management. First, plasma cell granuloma is an inflammatory pseudotumor presenting a reactive, non-neoplastic inflammatory lesion. The location of the lesions varies depending on the cases from solitary to systemically, but typically multiple. The histological appearance is mixed inflammatory infiltrate with abundant plasma cells, fibrosis, and mesenchymal cells. The pathogenesis of plasma cell granuloma is unknown but suggested that it has arisen through an immune-mediated inflammatory process [14]. Many plasma cell granulomas are now viewed as part of the IgG4-related disease spectrum, especially in the lung [15,16]. As shown in our case, infection is one of the leading backgrounds of plasma cell granuloma. Thus, the treatment modality is complete surgical excision, anti-inflammatory therapy, and/or antimicrobials.

Next, a plasma cell neoplasm is a tumor consisting of clonal plasma cell growth [17]. The clonality of the neoplasm is proven by the production of monoclonal immunoglobulin, as demonstrated by paraproteinemia involving gammaglobulin subclasses or light-chain restriction. Plasmacytoma is characterized by the pathological infiltration of atypical plasma cells in sheets or nodules, with minimal admixture of other inflammatory cells. The lesion is locally destructive and there is a risk of progression to systemic myeloma. Thus, treatment modalities include local radiotherapy and/or systemic anti-myeloma therapy. The most essential diagnostic distinction of plasma cell neoplasm from other reactive mass is proof of clonality [14]. Monoclonal gammaglobulinemia is one of the diagnostic criteria. Hypergammaglobulinemia alone, without clonal plasma cell expansion, is diagnosed as monoclonal gammopathy of undetermined significance (MGUS). Hypergammaglobulinemia accompanied by clonal plasma cell expansion is required to fulfill the diagnostic criteria for a plasma cell neoplasm. In our case, bone lesions and symptomatic pyrexia were also present, satisfying the clinical criteria for a diagnosis of plasma cell neoplasm.

Our experience through this case is a potentially rare situation. However, it would generalize the difficulty and importance of discriminating reactive pseudotumor from plasma cell neoplasm. The key discriminators of plasma cell neoplasm from plasma cell granuloma are below. (1) Clonality of plasma cells is essential. This should be confirmed with pathological sample. In bone marrow specimens, it should be more than 10% counts of plasma cells in all nucleic cells, which is objectively solid criteria. As a whole, in tissue specimen, it should be evaluated with unbalanced κ/λ light-chain restriction in immunochemistry staining but not in a count of cells. This finding was lacking in our case, although a proliferation of plasma cells was shown. In this point, inflammatory pathological changes are needed to be specifically differentiated with neoplastic architecture by a special pathologist. (2) Confirmation of monoclonality of gammaglobulinemia is a complementary factor to diagnose plasma cell neoplasm. We should remind that monoclonal proteins in serum/urine are merely corresponding findings. Shown in our case, this finding is sometimes tricky because some atypical cases of paraproteinosis in gammaglobulin are displayed, such as monoclonality in polyclonality, monoclonality without other gammaglobulin suppression, multiple monoclonality, etc.

## 4. Conclusions

The patient was finally discovered to have a bifurcated aortic aneurysm of infectious aortitis, as well as a sternal growing mass forming plasma cell granuloma and vertebral abscess. We learned from the case that the ^18^F-FDG-PET modality was useful to identify the biopsy site and determine the diagnosis by the disease localization.

## Figures and Tables

**Table 1:** Patient’s laboratory data at presentation.

Count of Blood Cells		
WBC	9580	/μL
Stab	0.0	%
Seg	90.5	%
Mon	3.5	%
Lym	4.5	%
Eos	1.5	%
Bas	0.0	%
RBC	286 × 10^4^	/μL
HGB	8.4	g/dL
HCT	25.4	%
MCV	88.8	fL
MCH	29.4	Pg
MCHC	33.1	%
RET	3.30	%
RET	9.44 × 10^4^	/μL
PLT	21.3 × 10^4^	/μL
IPF	11.5	%
Biochemistry		
CRP	0.86	mg/dL
TP	7.3	g/dL
ALB	3.2	g/dL
BUN	22.7	mg/dL
CRE	0.88	mg/dL
UA	5.8	mg/dL
T-BIL	0.5	mg/dL
AST	168	U/L
ALT	357	U/L
ALP	460	U/L
LDH	292	U/L
γGTP	45	U/L
sAmy	95	U/L
Na	135	mmol/L
K	4.8	mmol/L
Cl	104	mmol/L
Ca	8.6	mg/dL
IP	3.8	mg/dL
Mg	2.4	mg/dL
CPK	11	U/L
FBS	202	mg/dL
HbA1c	7.5	%
Fe	52	mg/dL
ferritin	687.0	ng/mL
sIL-2R	841	U/mL
Procalcitonin	0.08	ng/mL
Coagulofibrinolysis		
PT	70	%
PT-INR	1.18	
APTT	29.7	Sec
FIB	664	mg/dL
D-dimer	1.6	μg/mL
Immunochemistry		
IgA	434	mg/dL
IgG	2103	mg/dL
IgM	330	mg/dL
IgE	5013.6	IU/mL
IgG4	91.4	mg/dL
Serum IEP	(+)	
Urine IEP	(+)	

(Abbreviation: immunoelectrophoresis, IEP).

## Data Availability

All data generated or analyzed during this study are included in this published article. Data are available on reasonable request due to privacy and ethical restrictions.

## Abbreviations

The following abbreviations are used in this manuscript:

CT	Computed tomography
FDG-PET	^18^F-fluorodeoxyglucose positron emission tomography
MGUS	Monoclonal gammopathy of undetermined significance
